# Efficacy, Safety, Pharmacokinetics, and Immunogenicity of DRL-Trastuzumab Versus Herceptin in Human Epidermal Growth Factor Receptor 2–Positive Metastatic Breast Cancer: A Randomized Controlled Trial

**DOI:** 10.1200/GO.22.00328

**Published:** 2024-01-18

**Authors:** Naveen Reddy, Pramod Reddy, Ankit Ranpura, Narendra Maharaj, Rajendersingh Arora, Gopichand Mamillapalli, Aditya Shirish Adhav, Ashok Kumar Diwan, Alexey Manikhas, Dmitriy Krasnozhon

**Affiliations:** ^1^ Dr Reddy's Laboratories Ltd, Bachupally, India; ^2^ Sujan Surgical Cancer Hospital and Amravati Cancer Foundation, Amravati, India; ^3^ City Cancer Centre, Vijayawada, India; ^4^ Curie Manavata Cancer Centre, Mumbai Naka, Nashik, India; ^5^ Government Medical College and Hospital, Medical College Square, Nagpur, India; ^6^ St Petersburg State Budget Healthcare Institution “City Clinical Oncology Center”, St Petersburg, Russia; ^7^ State Budget Healthcare Institution “Leningrad Regional Oncology Center”, St Petersburg, Russia

## Abstract

**PURPOSE:**

Dr Reddy's Laboratories Trastuzumab (DRL_TZ) is a biosimilar to Herceptin under development. The present study was conducted to evaluate efficacy, safety, pharmacokinetics (PKs), and immunogenicity of DRL_TZ in comparison with the reference medicinal product (RMP) along with concomitant weekly paclitaxel in patients with human epidermal growth factor receptor 2 (HER2)–positive metastatic breast cancer (MBC).

**METHODS:**

This was a randomized, double-blind study in female patients with HER2-positive MBC, randomly assigned in a 1:1 ratio to receive either DRL_TZ or the RMP, that is, an innovator product sourced from the European region, along with additional chemotherapy, as first-line treatment for up to 24 weeks. The primary end point was the best overall response rate (ORR) as per RECIST 1.1 criteria. Progression-free survival rate at 6 months (PFS6), safety, immunogenicity, and PK parameters were assessed as secondary end points.

**RESULTS:**

A total of 164 patients were randomly assigned to receive either DRL_TZ or the RMP. Best ORR in the per-protocol population was comparable, 91.9% (93.3% CI, 83.2 to 96.3) versus 82.1% (93.3% CI, 72.0 to 89.1) in DRL_TZ and RMP arms, respectively; the difference between the arms was 9.8% with a 93.3% CI of –1.3 to 20.8. The PFS6 rate, safety, PK profile, and antidrug antibody incidence were comparable. An additional 44 patients were recruited in the postrandomization phase, in an open-label manner, and started on DRL_TZ to generate more data on efficacy, safety, and immunogenicity. The additional data with DRL_TZ, when pooled, were similar to the RMP data.

**CONCLUSION:**

DRL_TZ was found to have similar efficacy and comparable safety, PK, and immunogenicity profiles as the RMP.

## INTRODUCTION

Trastuzumab is a humanized monoclonal antibody of the IgG1 subtype that binds to human epidermal growth factor receptor 2 (HER2), activating antibody-dependent cellular cytotoxicity and blocking HER2 signaling.^1,2^ It is considered a standard therapy for HER2-positive early and advanced breast cancers and advanced gastroesophageal adenocarcinoma.^3-5^

**CONTEXT**

**Key Objective**
Dr Reddy's Laboratories Trastuzumab (DRL_TZ) is a biosimilar to Herceptin under development. DRL has developed a potential biosimilar for trastuzumab to Herceptin to improve affordability and patient access with comparable efficacy, safety, pharmacokinetics, and immunogenicity.
**Knowledge Generated**
Overall comparable efficacy was observed for best overall response rate (ORR), progression-free survival, and disease control rate with concomitant weekly paclitaxel in patients with human epidermal growth factor receptor 2–positive metastatic breast cancer (MBC). The safety profiles of DRL_TZ and the reference medicinal product (RMP), with respect to adverse events, treatment-emergent adverse events, and serious adverse events, were comparable (including infusion-related reactions) and aligned with the known safety profile. DRL_TZ demonstrated noninferiority in primary efficacy end point of best ORR as compared with the RMP when used as a first-line treatment in conjunction with paclitaxel and showed no remarkable differences in safety or immunogenicity profile, and hence, it is safely administered in patients with MBC.
**Relevance**
DRL has developed a potential biosimilar for trastuzumab to Herceptin to improve affordability and patient access with comparable efficacy, safety, pharmacokinetics, and immunogenicity.

Breast cancer is the most widespread cancer in women throughout the world. Incidence of breast cancer is increasing in developing countries, where the majority are diagnosed in late stages. In 2020, there were 2.3 million women diagnosed with breast cancer, causing 685,000 deaths globally.^6^ It is the most frequently reported cancer in women in India, with 178,361 newly diagnosed cases and 87,090 deaths in 2018.^7^

Increased price of novel cancer drugs is resulting in economic burden on low- and middle-income countries with limited patient access. Biosimilars in oncology can improve affordability and patient access.^8^ Dr Reddy's Laboratories (DRL) has developed a potential biosimilar for trastuzumab (DRL_TZ) to Herceptin (henceforth mentioned as the reference medicinal product [RMP]). Repeated dose toxicity studies were conducted in mice, primates (Cynomolgus monkeys), and local tolerance study in rabbits. The pharmacokinetic (PK) equivalence study in healthy volunteers demonstrated similarity, and no significant safety events were reported.^9^ This study was planned as a preapproval study for India and other emerging markets.

## METHODS

### Study Design

**FIG 1:**
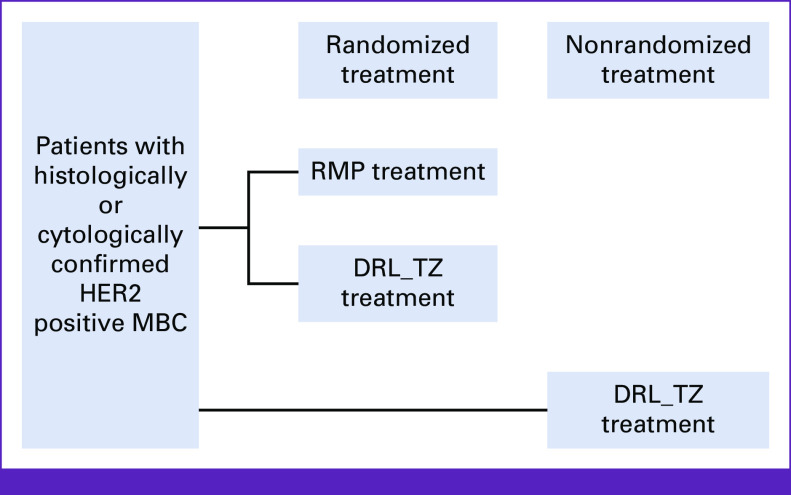
Study design. DRL_TZ, Dr Reddy's Laboratories Trastuzumab; HER2, human epidermal growth factor receptor 2; MBC, metastatic breast cancer; RMP, reference medicinal product.

This randomized, double-blind, multicenter, parallel-group study (Fig 1; CTRI/2015/08/006085) was conducted across 25 sites in India and Russia between September 2015 and May 2018. The study protocol was approved by the regulatory authorities of both countries and an independent ethics committee or institutional review board at each study center and conducted according to the Declaration of Helsinki, International Council for Harmonisation Good Clinical Practice guidelines, and applicable local regulations. All patients provided written informed consent before study participation.

### Patients

Eligible patients were females, were age between 18 and 75 years, had histologically or cytologically confirmed metastatic breast cancer (MBC), were treatment-naïve for MBC, and had at least one measurable lesion as per the RECIST 1.1 criteria.^10^ Study inclusion required a strong HER2-positive status validated by a +3 immunohistochemistry score, an Eastern Cooperative Oncology Group (ECOG) performance status of 0-2, a left ventricular ejection fraction (LVEF) of at least 50%, evaluated by echocardiography or multiple gated acquisition scan, and laboratory results in the protocol defined range.

The main exclusion criteria were history of any invasive malignancy except breast cancer or nonmelanoma skin cancer at least 1 year before random assignment, receipt of previous treatment for MBC or anti-HER2 therapy except when administered as (neo) adjuvant chemotherapy, active and/or history of cardiac disease, and cancer metastases in the central nervous system.

### Treatment

Eligible patients were randomly assigned on the basis of the interactive web-based response system to receive either DRL_TZ or the RMP in combination with paclitaxel for up to 24 weeks. Random assignment was stratified by previous use of trastuzumab in the (neo) adjuvant setting, menopausal status, and country.

Trastuzumab (DRL_TZ or RMP) was administered at an initial loading dose of 4 mg/kg body weight as an intravenous infusion over 90 ± 5 minutes (with adequate premedication), followed by once a week doses of 2 mg/kg administered by intravenous infusion over 30 ± 5 minutes. All patients received 80 mg/m^2^ once a week of paclitaxel as an intravenous infusion over 60 ± 10 minutes (with adequate premedication), after trastuzumab administration. The allowed premedications included paracetamol, pheniramine, granisetron, dexamethasone, ranitidine, hydrocortisone, and promethazine. The allowed concomitant medication included dexamethasone, ondansetron, and pantoprazole plus domperidone.

### End Points and Other Assessments

The primary end point was best overall response rate (ORR) assessed by the RECIST 1.1 criteria, measured by best response at any of the evaluable time points after random assignment until the end of the study (EOS), as evaluated by an independent blinded central radiology team to eliminate any subjective bias. The secondary efficacy end point was the evaluation of progression-free survival rate at 6 months (PFS6). The disease control rate (DCR) was evaluated as an exploratory end point. The DCR was defined as the proportion of patients who achieved complete response (CR) or partial response (PR) or stable disease (SD) up to week 25 or EOS. Other secondary end points included safety, PK evaluations (noncompartmental analysis or trough concentrations for selected subpopulations using Phoenix WinNonlin, Version 6.4), and incidence of antidrug antibodies (ADAs). On the basis of the established safety profile of trastuzumab, infusion-related reactions (IRRs) and the decrease in LVEF were also evaluated.

Tumor assessments by computed tomography or magnetic resonance imaging were performed during screening and at weeks 9, 17, and 25 (±1 week). In patients who discontinued prematurely for reasons other than progressive disease and for whom no radiographic assessments were available, a tumor assessment was performed at the EOS visit.

The patients who consented for PK sampling were divided into two subgroups: subgroup A of five patients and subgroup B of 15 patients, in each arm, respectively. Subgroup A patients were sampled for PK using an extended sampling schedule after the initial loading dose. Blood samples were collected preinfusion, 0.75 hours after the start of infusion, at 10 min after the end of infusion, and at 0.5 hour, 1 hour, 6 hours, 24 hours, 48 hours, and 72 hours after the end of infusion. Further samples were collected at week 2, week 6, week 12, and week 24 before trastuzumab administration. In Subgroup B patients, only trough samples were obtained before trastuzumab administration at weeks 1, 2, 6, 12, and 24.

Blood samples for immunogenicity analysis (ADA assessment) were collected before the first trastuzumab administration, at the start of week 6, and at week 25/EOS. In prematurely withdrawn patients, an immunogenicity sample was collected within 1 week after the last trastuzumab dose.

Safety assessments included treatment-emergent adverse events (TEAEs), alterations in LVEF and IRRs along with physical examination, 12-lead ECG changes, and laboratory abnormalities.

### Statistical Analysis

A sample size of 66 evaluable patients per treatment arm was estimated to provide a statistical power of 90% to rule out that the lower limit of the one-sided 95% CI (corresponding to a one-sided 90% CI after the application of the needed alpha-level adjustment for one interim analysis) for the ORR after DRL_TZ was below the ORR for the RMP, both along with once a week paclitaxel by more than the chosen noninferiority margin. The value of 18.50% was derived as half the difference in ORR observed between the trastuzumab plus paclitaxel group and the placebo plus paclitaxel group in the subgroup of HER2-positive 3+ patients in a published study.^11^

Considering an expected dropout rate of about 20% of patients, a sample size of 164 patients (82 in each arm) was considered appropriate. Ten patients in subgroup A and 30 patients in subgroup B were included for a descriptive comparison of the PK profiles. The pooled outcome of all DRL_TZ-exposed 126 patients (ie, 82 randomly assigned patients treated in a double-blind manner and the additional 44 patients treated in a nonrandomized and open-label manner) was presented separately.

The intention-to-treat (ITT) population for efficacy included all patients who were enrolled and randomly assigned to study treatment. The full analysis set/modified intention-to-treat (FAS/mITT) population was the subset of ITT population comprising patients who had at least one postbaseline tumor assessment. The per-protocol (PP) population included patients who received at least seven doses of study treatment, underwent the efficacy evaluations at week 9 (ie, week 9 visit), and did not have any major protocol deviations during the study participation that might affect efficacy analysis. Efficacy data are summarized as per the analyses conducted on the PP population (unless otherwise specified). The safety population included all the patients who received at least one dose of study drug. All patients who signed the informed consent form and for whom predose and postdose immunogenicity samples with valid results were available have been included for immunogenicity analysis. Immunogenicity was assessed at the central laboratory, Syngene International Limited, using validated methods. Immunogenicity was initially tested by a screening assay, and those who were identified positive were retested using confirmatory assays. Confirmatory assay positives were considered as ADA positives, and those considered ADA positives by confirmatory assay were tested using neutralizing antibody assay.

Continuous data are summarized with the number of observations, mean, standard deviation, median, minimum and maximum, and geometric mean and its coefficient of variation. Categorical data were summarized by treatment arm using frequency tables (frequencies and percentages).

The primary end point was analyzed by calculating CIs for the difference in best ORR between the DRL_TZ and the RMP arm using the Wilson Score method and the following type I error adjustments for the interim analyses: three primary end point analyses, interim analyses (to reconfirm sample size and to fulfill the regulatory requirement), and a final analysis were performed. Hence, at the end of the study, a correction using the Bonferroni method of the full study type I error level (one-sided α = .10) for two interim analyses was applied and α was adjusted to (one-sided α)/3 = one-sided alpha .033 for final analysis. Final analysis primary end point results were concluded at one-sided α = .033 (two-sided alpha of .067 corresponding to a 93.3% CI) to preserve an overall type I error rate for the study of one-sided α = .10. The same analysis was applied for the secondary end point PFS6 and the exploratory end points ORR and DCR.

## RESULTS

**FIG 2:**
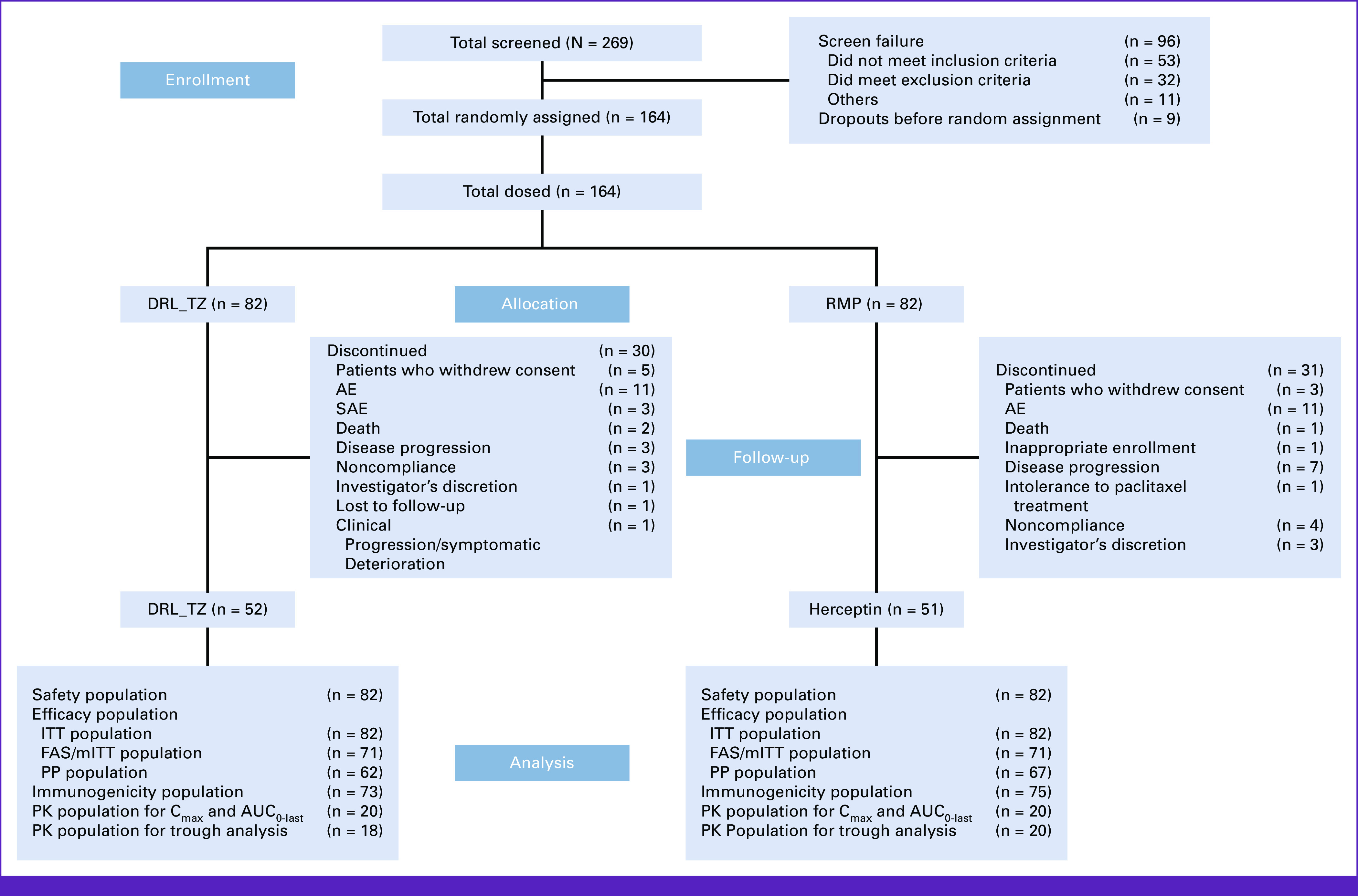
Patient disposition and analysis population (randomly assigned patients). AE, adverse event; AUC_0-last_, area under the concentration-time curve from zero (0) hours to the last time point; C_max_, maximum concentration of drug; DRL_TZ, Dr Reddy's Laboratories Trastuzumab; FAS, full analysis set; ITT, intention-to-treat; mITT, modified ITT; PK, pharmacokinetic; PP, per protocol; RMP, reference medicinal product; SAE, serious adverse event.

Of the 269 patients screened, 164 patients were found to be suitable for inclusion in the study. These patients were randomly assigned in a 1:1 ratio to receive either DRL_TZ or the RMP for up to 24 weeks. Among these, 52 patients on DRL_TZ and 51 patients on the RMP completed the study. Patient disposition is presented in Figure 2.

To comply with the Guideline on Similar Biologics—Regulatory Requirement for Marketing Authorization in India 2016, an additional 44 patients were recruited to the DRL_TZ arm, which ensured at least 100 patient exposure to the DRL_TZ test arm.^12^ The study procedures and assessments for these additional 44 patients were similar to those for the earlier recruited patients except being unblinded and nonrandomized. Data are presented for the randomly assigned patients followed by pooled results of all DRL_TZ-exposed patients (n = 126, ie, 82 randomly assigned plus additional 44 not randomly assigned patients) under the heading “Pooled test arm patients.”

### Demographics and Baseline Characteristics

Age, race, weight, BMI, and ECOG performance status were balanced between the treatment arms. Majority patients were Asian (90.2%; Table 1).

**TABLE 1:** Patient Demographics and Baseline Characteristics—Safety Population

Demographic	Randomly Assigned Patient			Pooled Test Arm Patient^a^
	DRL_TZ (n = 82)	RMP (n = 82)	Total (n = 164)	DRL_TZ (n = 126)
Age,^b^ years, mean (±standard deviation)	50.37 (10.95)	49.04 (10.40)	49.70 (10.67)	50.48 (10.57)
Race, No. (%)				
Asian	74 (90.2)	74 (90.2)	148 (90.2)	118 (93.7)
White-White/White/European heritage	8 (9.8)	8 (9.8)	16 (9.8)	8 (6.3)
Weight, kg, mean (±standard deviation)	57.14 (11.68)	58.26 (12.22)	57.70 (11.93)	57.15 (12.48)
BMI, kg/m^2^,^c^ mean (±standard deviation)	24.29 (3.89)	24.81 (5.11)	24.55 (4.54)	24.37 (4.35)
ECOG performance status, No. (%)				
0	22 (26.8)	20 (24.4)	42 (25.6)	31 (24.6)
1	59 (72.0)	62 (75.6)	121 (73.8)	94 (74.6)
2	1 (1.2)	0	1 (0.6)	1 (0.8)

Abbreviations: DRL_TZ, Dr Reddy's Laboratories Trastuzumab; ECOG, Eastern Cooperative Oncology Group; RMP, reference medicinal product.

^a^ Pooled test arm includes data of 82 randomly assigned patients on DRL_TZ and additional 44 nonrandomized patients on DRL_TZ.

^b^ Age (years) = (date of the screening visit – date of birth)/365.25.

^c^ BMI (kg/m^2^) = weight (kg)/(height [cm] × height [cm]).

### Efficacy

For the ITT population up to week 25, CR was observed in five patients in the DRL_TZ arm and in one patient in the RMP arm. While 40 patients in the DRL_TZ arm and 37 patients in the RMP arm achieved a PR, SD was observed in two and three patients in the DRL_TZ arm and in the RMP arm, respectively (Table 2). The FAS/mITT and PP population also showed very similar data.

**TABLE 2:** Summary of ORR at Different Time Points as Measured by Central Imaging

Population	Final Time Point ORR										
			Week 9			Week 17			Week 25		
			Randomly Assigned Patient		Pooled Test Arm Patient^a^	Randomly Assigned Patient		Pooled Test Arm Patient^a^	Randomly Assigned Patient		Pooled Test Arm Patient^a^
	Type	Statistic	DRL_TZ (n = 82)	RMP (n = 82)	DRL_TZ (n = 126)	DRL_TZ (n = 82)	RMP (n = 82)	DRL_TZ (n = 126)	DRL_TZ (n = 82)	RMP (n = 82)	DRL_TZ (n = 126)
ITT	CR	No. (%)	1 (1.2)	0	1 (0.8)	2 (2.4)	0	3 (2.4)	5 (6.1)	1 (1.2)	5 (4.0)
	PR	No. (%)	49 (59.8)	46 (56.1)	79 (62.7)	49 (59.8)	50 (61.0)	72(57.1)	40 (48.8)	37 (45.1)	59 (46.8)
	SD	No. (%)	12 (14.6)	16 (19.5)	17(13.5)	3 (3.7)	4 (4.9)	6 (4.8)	2 (2.4)	3 (3.7)	2 (1.6)
	PD	No. (%)	2 (2.4)	5 (6.1)	4 (3.2)	2 (2.4)	5 (6.1)	2 (1.6)	3 (3.7)	7 (8.5)	5 (4.0)
FAS/mITT	Type	Statistic	DRL_TZ (n = 71)	RMP (n = 71)	DRL_TZ (n = 110)	DRL_TZ (n = 71)	RMP (n = 71)	DRL_TZ (n = 110)	DRL_TZ (n = 71)	RMP (n = 71)	DRL_TZ (n = 110)
	CR	No. (%)	1 (1.4)	0	1 (0.9)	2 (2.8)	0	3 (2.7)	5 (7.0)	1 (1.4)	5 (4.5)
	PR	No. (%)	49 (69.0)	46 (64.8)	79 (71.8)	49 (69.0)	50 (70.4)	72 (65.5)	40 (56.3)	37 (52.1)	59 (53.6)
	SD	No. (%)	12 (16.9)	16 (22.5)	17 (15.5)	17 (15.5)	4 (5.6)	6 (5.5)	2 (2.8)	3 (4.2)	2 (1.8)
	PD	No. (%)	2 (2.8)	5 (7.0)	4 (3.6)	4 (3.6)	5 (7.0)	2 (1.8)	3 (4.2)	7 (9.9)	5 (4.5)
PP	Type	Statistic	DRL_TZ (n = 62)	RMP (n = 67)	DRL_TZ (n = 98)	DRL_TZ (n = 62)	RMP (n = 67)	DRL_TZ (n = 98)	DRL_TZ (n = 62)	RMP (n = 67)	DRL_TZ (n = 98)
	CR	No. (%)	1 (1.6)	0	1 (1.0)	1 (1.6)	0	2 (2.0)	4 (6.5)	1 (1.5)	4 (4.1)
	PR	No. (%)	47 (75.8)	45 (67.2)	76 (77.6)	45 (72.6)	48 (71.6)	66 (67.3)	37 (59.7)	36 (53.7)	54 (55.1)
	SD	No. (%)	11 (17.7)	16 (23.9)	16 (16.3)	3 (4.8)	4 (6.0)	6 (6.1)	2 (3.2)	3 (4.5)	2 (2.0)
	PD	No. (%)	2 (3.2)	5 (7.5)	4 (4.1)	2 (3.2)	4 (6.0)	2 (2.0)	2 (3.2)	7 (10.4)	4 (4.1)

Abbreviations: CR, complete response; DRL_TZ, Dr Reddy's Laboratores Trastuzumab; FAS, full analysis set; ITT, intention-to-treat; mITT, modified ITT population; ORR, overall response rate; PD, progressive disease; PP, per protocol; PR, partial response; RMP, reference medicinal product; SD, stable disease.

^a^ Pooled test arm includes data of 82 randomly assigned patients on DRL_TZ and additional 44 nonrandomized patients on DRL_TZ.

In the primary efficacy analysis, the best ORR was 91.9% (93.3% CI, 83.2 to 96.3) for the DRL_TZ arm (n = 62) and 82.1% (93.3% CI, 72.0 to 89.1) for the RMP arm (n = 67). The difference in proportion between the DRL_TZ and RMP arms was 9.8% with a two-sided 93.3% CI of –1.3 to 20.8 (Table 3). The FAS/mITT and PP population also showed very similar data.

**TABLE 3:** Best Overall Response Rate, CR + PR

Analysis Population	Statistic	Randomly Assigned Patient			Pooled Test Arm Patient^a^
		DRL_TZ		RMP	DRL_TZ
	Total patients	n = 82		n = 82	n = 126
ITT	Patients with response, No. (%)	64 (78.0)		57 (69.5)	98 (77.8)
	Two-sided 93.3% CI (one-sided 96.7% CI): (LCL, UCL)^b^	68.7, 85.2		59.6, 77.9	70.3, 83.8
	Difference in percentage—Wilson score method (%)		8.5		NA
	Two-sided 93.3% CI (one-sided 96.7% CI): (LCL, UCL)%^c^	–4.1, 20.8			NA
FAS/mITT	Total patients	n = 71		n = 71	n = 110
	Patients with response, No. (%)	64 (90.1)		57 (80.3)	98 (89.1)
	Two-sided 93.3% CI (one-sided 96.7% CI): (LCL, UCL)^b^	81.7, 94.9		70.4, 87.5	82.4, 93.4
	Difference in percentage—Wilson score method (%)		9.9		NA
	Two-sided 93.3% CI (one-sided 96.7% CI) for difference in percentage: (LCL, UCL)%^c^	–1.2, 20.9			NA
PP	Total patients	n = 62		n = 67	n = 98
	Patients with response, No. (%)	57 (91.9)		55 (82.1)	88 (89.8)
	Two-sided 93.3% CI (one-sided 96.7% CI): (LCL, UCL)^b^	83.2, 96.3		72.0, 89.1	82.8, 94.1
	Difference in percentage—Wilson score method (%)		9.8		NA
	Two-sided 93.3% CI (one-sided 96.7% CI) for difference in percentage: (LCL, UCL)%^c^	–1.3, 20.8			NA

NOTE. Percentages are calculated of total patients in the respective population (No.).

Abbreviations: CR, complete response; DRL_TZ, Dr Reddy's Laboratories Trastuzumab; FAS, full analysis set, ITT, intention to treat; LCL, lower confidence limit; mITT, modified ITT population; PP, per protocol; PR, partial response; RMP, reference medicinal product; UCL, upper confidence limit.

^a^ Pooled test arm includes data of 82 randomly assigned patients on DRL_TZ and additional 44 nonrandomized patients on DRL_TZ.

^b^ Two-sided 93.3% CIs (one-sided 96.7% CI) for one sample proportion are estimated using the Wilson score method.

^c^ Two-sided 93.3% CIs (one-sided 96.7% CI) for difference in two independent proportions are estimated using the Wilson score method.

Progression-free survival for the PP population at 6 months was observed in 41 patients (PFS6 rate, 85.4% [two-sided 93.3% CI, 73.8 to 92.4]) in the DRL_TZ arm and in 39 patients (PFS6, 70.9% [two-sided 93.3% CI, 58.8 to 80.7]) in the RMP arm. The difference in PFS6 rate between arms was 14.5% with a two-sided 93.3% CI of –0.7 to 28.6 (Table 4). Disease control as best overall response data for the PP population on the DRL_TZ arm was observed in 60 patients (96.8% [two-sided 93.3% CI, 89.7 to 99.0]), and that on the RMP arm was observed in 61 patients (91.0% [two-sided 93.3% CI, 82.6 to 95.6]). The difference in percentage between the arms was 5.7 with a 93.3% CI of –2.7 to 14.5 (Table 4).

**TABLE 4:** Summary of PFS6 and DCR (PP population)

Efficacy Parameter (PP population)	Randomly Assigned Patient		Difference With Two-Sided 93.3% CI (one-sided 96.7% CI)	Pooled Test Arm Patient^a^
	DRL_TZ (n = 62)	RMP (n = 67)		DRL_TZ (n = 98)
PFS6				
No. of patients who had PFS6	48	55		70
Patients with response, No. (%)	41 (85.4)	39 (70.9)		57 (81.4)
Two-sided 93.3% CI (one-sided 96.7% CI)	73.8 to 92.4	58.8 to 80.7		71.6 to 88.4
			14.5 (–0.7 to 28.6)	
DCR using BOR: CR + PR + SD				
Patients with response, No. (%)	60 (96.88)	61 (91.0)		94 (95.9)
Two-sided 93.3% CI (one-sided 96.7% CI): (LCL, UCL)^b^	89.7, 99.0	82.6, 95.6		90.5, 98.3
			5.7 (–2.7 to 14.5)^c^	

Abbreviations: CR, complete response; DCR, disease control rate; LCL: Lower Confidence Limit, PFS6: Progression-Free Survival at the end of 6 months, PR, partial response; RMP, reference medicinal product; SD, stable disease; UCL: Upper Confidence Limit.

^a^ Pooled test arm includes data of 82 randomly assigned patients on DRL_TZ and additional 44 nonrandomized patients on DRL_TZ.

^b^ Two-sided 93.3% CIs (one-sided 96.7% CI) for one sample proportion are estimated using the Wilson score method.

^c^ Two-sided 93.3% CIs (one-sided 96.7% CI) for difference in two independent proportions are estimated using the Wilson score method.

Overall comparable data were observed in the randomly assigned patients and pooled DRL_TZ patients (Table 4) for best ORR, PFS, and DCR.

### Safety

In total, 155 patients experienced TEAEs, including 78 (95.1%) patients and 77 (93.9%) patients in the DRL_TZ arm and the RMP arm, respectively (Table 5). The most frequently reported TEAEs (>10% in either arm) in the DRL_TZ and RMP arms were anemia, alopecia, asthenia, diarrhea, peripheral sensory neuropathy, leukopenia, pyrexia, cough, peripheral neuropathy, and pain. The most frequently reported study drug–related TEAEs were asthenia in six (7.3%) patients in the DRL_TZ arm and three (3.7%) patients in the RMP arm, nausea in five (6.1%) patients in the DRL_TZ arm and three (3.7%) patients in the RMP arm, and pain in four (4.9%) patients in the DRL_TZ arm and one (1.2%) patient in the RMP arm. Thirty-four serious adverse events (SAEs) were reported in 23 patients during the study including 20 SAEs in 13 (15.9%) patients in the DRL_TZ arm and 14 SAEs in 10 (12.2%) patients in the RMP arm. Three patients experienced fatal events, including one event each of sudden death and disease progression in the DRL_TZ arm and a fatal event of respiratory distress in the RMP arm. None of these fatal SAEs were related to the study drug. All other nonfatal events got resolved during the study. Thirty (36.6%) patients in the DRL_TZ arm and 33 (40.2%) patients in the RMP arm experienced TEAEs of severity grade 3 or above.

**TABLE 5:** Overview of Treatment-Emergent Adverse Events by Treatment Arm—Safety Population

TEAE	Randomly Assigned Patient		Total (n = 164), No. (%)	Pooled Test Arm Patient^a^
	DRL_TZ (n = 82), No. (%)	RMP (n = 82), No. (%)		DRL_TZ (n = 126), No. (%)
Any AE	78 (95.1)	77 (93.9)	155 (94.5)	121 (96.0)
Grade 3 or higher-intensity AE	30 (36.6)	33 (40.2)	63 (38.4)	52 (41.3)
AE related to study treatment	28 (34.1)	18 (22.0)	46 (28.0)	38 (30.2)
Grade 3 or higher intensity-related AE	6 (7.3)	1 (1.2)	7 (4.3)	12 (9.5)
AE leading to permanent discontinuation of study drug or withdrawal from the study	17 (20.7)	17 (20.7)	34 (20.7)	25 (19.8)
Any nonserious AE	78 (95.1)	74 (90.2)	152 (92.7)	121 (96.0)
Grade 3 or higher-intensity AE	26 (31.7)	30 (36.6)	56 (34.1)	45 (35.7)
AE related to study treatment	27 (32.9)	17 (20.7)	44 (26.8)	37 (29.4)
Grade 3 or higher intensity-related AE	3 (3.7)	0	3 (1.8)	9 (7.1)
AE leading to permanent discontinuation of study drug or withdrawal from the study	12 (14.6)	15 (18.3)	27 (16.5)	17 (13.5)
Any SAE	13 (15.9)	10 (12.2)	23 (14.0)	19 (15.1)
Grade 3 or higher-intensity SAE	10 (12.2)	6 (7.3)	16 (9.8)	15 (11.9)
SAE related to study treatment	3 (3.7)	1 (1.2)	4 (2.4)	3 (2.4)
Grade 3 or higher intensity-related SAE	3 (3.7)	1 (1.2)	4 (2.4)	3 (2.4)
Fatal SAE not related to study treatment	2 (2.4), 2	1 (1.2)	3 (1.8)	4 (3.2)
Fatal SAE related to study treatment	0	0	0	0
SAE leading to permanent discontinuation of study drug or withdrawal from the study	5 (6.1)	2 (2.4)	7 (4.3)	8 (6.3)

Abbreviations: AEs, adverse events; RMP, reference medicinal product; SAEs, serious adverse events; TEAE, treatment-emergent adverse event.

^a^ Pooled test arm includes data of 82 randomly assigned patients on DRL_TZ and additional 44 nonrandomized patients on DRL_TZ.

Regarding IRRs, five (6.1%) patients in the DRL_TZ arm reported chills and two (2.4%) patients in the RMP arm reported chills. One patient per arm reported pyrexia, all in mild (grade 1) intensity. These led to temporary interruption of drug infusion (except one event in the DRL_TZ arm where there was no such dose interruption) and were resolved completely.

Six patients in the DRL_TZ randomized arm (four [4.9%] patients with decreased ejection fraction and two [2.4%] patients with left ventricular dysfunction) and three (3.7%) patients (decreased ejection fraction) in the RMP arm experienced cardiac toxicity. A total of 10 cardiotoxicity events were reported in 10 patients in the pooled DRL_TZ test arm (eight events of ejection fraction decreased [6.3%], two events of left ventricular dysfunction [1.6%]). In addition, three (3.7%) patients experienced hypertension and one event of angina pectoris in the RMP arm. None of these events were reported with DRL_TZ. The investigator is obligated to assess the relationship between the study drugs and the occurrence of each AE or SAE with the clinical judgment, consulting the protocol and product brochure. Alternative causes, such as natural history of the underlying diseases, concomitant chemotherapy, other risk factors, and the temporal relationship of the event to study drug administration, were considered and investigated.

### PKs

The PK population consisted of all patients who were enrolled, randomly assigned to the study treatment DRL_TZ/RMP, the primary PK parameters reliably calculated and have completed the study without major protocol deviations which may significantly affect the PK of the drug. All patients for whom at least four PK samples were available with a recorded time and a result for the study drug concentration reported by the bioanalytical laboratory were analyzed in the PK population. For trough analysis, all patients from subgroup A and subgroup B were included, for whom a PK sample with a recorded time obtained after the administration of first dose of study drug and before the administration of subsequent weekly dose of the study drug and a result for study drug concentration reported by the bioanalytical laboratory were available. PK parameters maximum concentration of drug (C_max_), area under the concentration-time curve from zero (0) hours to the last time point (AUC_0-last_), and the trough concentration (C_trough_) values for DRL_TZ and Herceptin were estimated and presented descriptively. The trastuzumab PK parameters were comparable for both treatment arms (Table 6).

**TABLE 6:** PK Parameters

PK Parameter, Unit	No.		Randomly Assigned Patient		
			DRL_TZ (n = 18)	No.	RMP (n = 20)
C_max_, μg/mL, mean (±standard deviation)	4		110.739 (218.2479)	5	101.158 (32.274)
AUC_0-last_, μg × h/mL, mean (±standard deviation)	4		7,779.988 (±1,925.90)	4	8,637.08314 (2017.945)
C_trough_ (CV %), geometric mean	18	C_t2_	25.884 (0.051)	20	27.684 (0.020)
	16	C_t6_	42.369 (0.031)	19	37.336 (0.037)
	14	C_t12_	58.204 (0.074)	17	46.734 (0.032)
	11	C_t24_	58.246 (0.044)	13	53.115 (0.056)

Abbreviations: AUC_0-last_, area under the concentration-time curve from zero (0) hours to the last time point; C_max_, maximum concentration of drug; C_trough_, trough concentration; CV, coefficient of variation; DRL_TZ, Dr Reddy's Laboratories Trastuzumab; PK, pharmacokinetic; RMP, reference medicinal product.

### Immunogenicity

One patient (1.4%) assigned to DRL_TZ tested positive for antidrug antibodies (neutralizing in the specific assay) before dosing. This patient tested negative for ADAs at the EOS. Three patients (one in DRL_TZ at week 25 and two in the RMP arm both at week 6) tested positive for ADA postdose. None of these ADAs were neutralizing.

In the pooled DRL_TZ arm, in addition to the earlier mentioned patient, two additional patients tested positive for ADA postdose at week 6 and week 25, respectively, although both these patients were negative to neutralizing ADA at EOS visits. Hence, it was concluded that the incidence of ADA is low and comparable and no neutralizing ADAs were observed in either of the treatment arms.

## DISCUSSION

This randomized double-blind study compared the efficacy, safety, immunogenicity, and PK of DRL_TZ with the RMP in patients with advanced HER2-positive breast cancer.

The ORR is considered a reliable end point for estimating the product-related differences between the reference and test drug as it is a direct measure of the drug’s antitumor activity.^13^ The best ORR over 24 weeks was statistically compared with two-sided 93.3% CI for the difference in proportions between the DRL_TZ and the RMP arm. The best ORR in the PP population in the DRL_TZ arm was 91.9%, and in the RMP arm, it was 82.1%. The difference in percentage between both arms was 9.8% with a 93.3% CI of –1.3 to 20.8. The lower 93.3% CI for the difference between arms is above the protocol-defined noninferiority limit of –18.5%, proving the noninferiority of DRL_TZ’s efficacy versus the RMP. Thus, the primary objective of this study was met. Results were comparable for the FAS/mITT population and the ITT population, further proving noninferiority of DRL_TZ over the RMP. The ORR observed was in line with that observed in the literature with RMP phase II/III clinical trials in patients with HER2-positive advanced breast cancer (70%-81%).^14-16^

These results were also in line with the study conducted by Rugo et al comparing another potential trastuzumab biosimilar with the RMP in patients with MBC along with paclitaxel or docetaxel up to week 24. The observed ORR at week 24 was 69.6%, and the calculated DCR was 85.5%.^16^

The progression-free survival rate at 6 months (85.4% in the DRL_TZ arm, 70.9% in the RMP arm for the PP population) was comparable in both arms, and no statistically significant differences were observed between treatment arms.

The safety profiles of DRL_TZ and the RMP, with respect to AEs, TEAEs, and SAEs, were comparable (including IRRs) and aligned with the known safety profile of trastuzumab. Because of the potential cardiotoxicity of trastuzumab, we closely examined the LVEF and any other signs of cardiac dysfunction. A low incidence of cardiotoxicity (<10%) was observed in both treatment groups throughout the study.

The trastuzumab PK parameters were comparable for both treatment arms. A statistical comparison with descriptive purpose did not show any significant differences (the 1.000 equality indicating value for the ratio lies within the 90% CI), supporting PK biosimilarity. Similarly, C_trough_ values in both treatment arms were comparable at the protocol-defined evaluation times.

The incidence of treatment-emergent ADAs in the study was low (one patient in the DRL_TZ arm; two patients in the RMP arm, and one [1.4%] patient in the open-label DRL_TZ test arm), and the detected treatment-emergent ADAs had no neutralizing capacity, consistent with low immunogenicity as reported in the literature.^14,15,17,18^

Demonstrating clinical efficacy and safety in comparative clinical studies is the last step in the development of biosimilars.^19^ On the basis of the results, it was concluded that DRL_TZ demonstrated noninferiority in the primary efficacy end point of ORR as compared with the RMP when used as a first-line treatment in conjunction with paclitaxel and showed no remarkable differences in safety or immunogenicity profile. Moreover, 90% CIs for the differences in PFS6 and DCR showed no significant difference and the evaluation of all the 126 patients in the test arm (DRL_TZ) showed comparable results with those in the randomized DRL_TZ arm, which supports the robustness of the conclusions.
