# Erratum: Efficacy, Safety, Pharmacokinetics, and Immunogenicity of DRL-Trastuzumab Versus Herceptin in Human Epidermal Growth Factor Receptor 2–Positive Metastatic Breast Cancer: A Randomized Controlled Trial

**DOI:** 10.1200/GO.24.00076

**Published:** 2024-03-07

The article by Reddy et al entitled “Efficacy, Safety, Pharmacokinetics, and Immunogenicity of DRL-Trastuzumab Versus Herceptin in Human Epidermal Growth Factor Receptor 2–Positive Metastatic Breast Cancer: A Randomized Controlled Trial” (*JCO Global Oncology* 10.1200/GO.22.00328) was published online January 18, 2024, with an error.

The acknowledgments section provided was incomplete. The full text should read:

The authors thank Dr Sonica S. Batra, Dr Aseem Srivastava, Sidharth Bichpuria, Kamala Bhavaraju, Dr Vikas Kumar, Amrita Chowdhury for their contribution to the study conduct, critical review of the data, and manuscript preparation.

The authors also thank Dr Radheshyam Naik, Dr Rosina Ahmed, Dr Rakesh Chopra, Dr Naseem Akhtar, Dr Divya Bala Thumathy, Dr Adwaita Anant Gore, Dr Mamraj Gupta, Dr Nilesh Mahale, Dr Padmaj Kulkarni, Dr Ajaykumar Thakaji Jadhav, Dr Viraj Borgaonkar, Dr Krishnan Srinivasan, Dr Muralikrishna Voonna, Dr Radhika Parimkayala, Dr Akila K B, Dr Bhavin Ashok Bhai Shah, the investigators in Trastuzumab study group for their contribution to the study and Dr Shona Milon Nag who acted as Data and Safety Monitoring Board Chair along with members Dr Vineet Gupta, Dr K.K. Sharma, Jaishree Ganjiwale.

This has been corrected as of March 7, 2024. The authors apologize for the error.

